# National trend in heart failure hospitalization and outcome under public health insurance system in Thailand 2008–2013

**DOI:** 10.1186/s12872-022-02629-2

**Published:** 2022-04-29

**Authors:** Satit Janwanishstaporn, Khemajira Karaketklang, Rungroj Krittayaphong

**Affiliations:** 1 Department of Medicine, Faculty of Medicine Siriraj Hospital, Mahidol University, Bangkok, Thailand; 2 Division of Cardiology, Department of Medicine, Siriraj Hospital, Mahidol University, 2 Wanglang Road Bangkoknoi, Bangkok, 10700 Thailand

**Keywords:** National trend, Heart failure, Hospitalization, Thailand

## Abstract

**Background:**

There are limited data on the burden, characteristics, and outcomes of hospitalized heart failure (HF) patients in Thailand. The aim of this study was to investigate national trend in HF hospitalization rate, in-hospital and 1-year mortality rate, and rehospitalization rate in Thailand.

**Methods:**

We analyzed the claims data of hospitalized patients obtained from the three major Thailand public health reimbursement systems between 2008 and 2013. Patients aged ≥ 18 years with a principal diagnosis of HF by the International Classification of Diseases, Tenth Revision, Thai modification were included. Comorbidities were identified by secondary diagnosis codes. The annual rate of HF hospitalization was calculated per 100,000 beneficiaries. Records of subsequent hospitalization of discharged patients were retrieved. For 1-year mortality rate, vital status of each patient was obtained from Thai Civil Registration of Death database. All outcomes were tested for linear trends across calendar years.

**Results:**

Between 2008 and 2013, 434,933 HF hospitalizations were identified. The mean age was 65.3 years (SD 14.6), and 58.1% were female. The HF hospitalization rate increased from 138 in 2008 to 168 per 100,000 beneficiaries in 2013 (*P* for trend < 0.001). Nearly half (47.4%) had had a prior HF admission within 1 year. A small proportion of patients (7.4%) received echocardiography during hospitalization. The median length of hospital stay was 3 days. In-hospital mortality declined from 4.4 to 3.8% (*P* for trend < 0.001). The overall 30-day and 1-year rehospitalization rates were 34 and 73%, respectively, without significant trends over the study period. Most common cause of 30-day rehospitalization was HF (42%). One-year mortality decreased from 31.8% in 2008 to 28.5% in 2012 (*P* for trend < 0.001).

**Conclusion:**

Between 2008 and 2013, HF hospitalization rate in Thailand increased. The in-hospital and 1-year mortality rates decreased slightly. However, the rehospitalization rate remained high mainly due to recurrent HF hospitalization.

**Supplementary Information:**

The online version contains supplementary material available at 10.1186/s12872-022-02629-2.

## Introduction

Heart failure (HF) is increasingly becoming a major health problem in many countries. One of the issues is HF hospitalization. Because HF is a common hospitalized condition in aging populations with a high readmission rate and long-term mortality risk [1, 2], it consumes a significant amount of health budget from both direct and indirect costs [3]. However, epidemiological data of this condition are limited in low- and middle-income countries (LMICs), including Thailand [4]. In addition, there are differences in the epidemiology, characteristics, and prognosis of hospitalized HF patient between countries in different world regions [5]. Until now, ADHERE-Thailand is the only sizable registry of hospitalized HF patients for the country [6]. However, the registry only collects data in cardiac centers in tertiary hospitals or large private hospitals, mostly in the Bangkok metropolitan area. Therefore, their data might not represent the overall picture of nationally for hospitalized patients with HF. Also, this registry has not assessed the long-term outcomes of these patients.

As the health care provision for the Thai population has been improving since the implementation of universal health coverage system for all Thai people in 2002 [7], HF and other conditions that can cause HF, such as hypertension and myocardial infarction, might have been treated more effectively [8]. Thus, the hospitalization rate of HF is supposed to have decreased and prognosis of HF hospitalization is supposed to have improved. Conversely, the number of older adults in Thailand has been increasing [9], and survivors from cardiovascular disease (e.g., acute myocardial infarction without appropriate treatment) might develop HF [8]. Both of these factors may increase the rate of HF hospitalization. To date, the rate and trend of HF hospitalization in Thailand are unknown. Understanding the health burden of HF is necessary to prevent and improve the outcomes of this lethal condition for the future aging society of Thailand.

Thus, we aimed to determine the national HF hospitalization rate, and the characteristics of hospitalized HF patients in Thailand from 2008 to 2013 using public health security scheme data. We also aimed to determine the rates of rehospitalization and mortality of hospitalized HF patients, and the factors associated with them.

## Methods

### Study design, setting, and sources of data

This was a nationwide, retrospective observational study using in-patient medical expense claims data to identify adult patients hospitalized with HF from 2008 to 2013 in Thailand. The Siriraj Institutional Review Board reviewed the protocol and approved the study with an exemption from requiring consent due to the retrospective nature of the study (ethics committee code 647/2556). All patient identities were encrypted from the data sources. The study followed the Reporting of Studies Conducted Using Observational Routinely-collected Health Data guideline [10].

We retrieved the in-patient electronic health medical expense claims data from three major public health security schemes covering all Thai citizens of all ages. Each citizen is allowed to have only one public health security coverage at any given time. These three schemes are the Civil Servant Medical Benefit scheme (CSMBS) for 4.4 million government officers and their families, the Social Security Scheme (SSS) for 10.6 million employees in the private sector, and the Universal Health Coverage scheme (UCS), which has covered the remaining 48 million persons since 2002 [7]. The public and participating private hospitals throughout Thailand send the electronic discharge records of each hospitalized patient to the online claims reimbursement system of one of these schemes. The amount of payment for hospitalized patient reimbursement is determined by Diagnostic-Related Groups version 5.0 (Office of the Development of Thai Joint Disease Group, Institute of Health Systems Research Institute, Thailand) calculated from each record data. These data include the patient’s unique identifier, age, sex, national identification number (ID), hospital identifier, admission and discharge dates, discharge status, primary diagnosis and secondary diagnoses coded in the International Classification of Diseases, Tenth Revision, Thai modification (ICD-10 TM code), procedure coded in the International Classification of Diseases, Ninth Revision, Clinical Modification (ICD-9 CM), and the amount of hospital expenses charged. The Bureau of Claims and Medical Audit (BCMA) of National Health Service Office (NHSO) periodically audits selected claims medical records for data quality and accuracy.

### Participants, data access, and data linkage

We requested the data centers of the three public health security schemes to retrieve the medical claims data of all patients aged ≥ 18 years with HF hospitalization as the principal diagnosis from 2008 to 2013, referred to as the index HF hospitalization. The ICD-10 TM codes for HF were I50.0, I50.1, I50.9, I11.0, I13.0, and I13.2. Next, the records of all hospitalization (with any principal diagnosis) from 2007 to 2013 of corresponding HF patient were identified for analysis of comorbidities and rehospitalizations. Exclusion criteria were erroneous data entries (e.g., very extreme age or duplicate admissions) or overlapping admission periods in the same patient without the code of the patient’s transfer.

For vital status and date of death, each data center linked the patient’s national ID with the Thai Civil Registration of Death (TCRD) from the Bureau of Registration administration of Thailand. The vital statuses of the patients were followed until the 31 December 2013 in the CSMBS and SSS and 26 June 2014 in the UCS. After retrieving the linked information, all patient identifiers were encrypted before the data were sent to the investigators.

### Patient comorbidities and procedures

The comorbidities and complications were defined by the ICD-10 TM in secondary diagnosis codes in the claim records of the index HF hospitalizations, which allowed for a maximum of 20 codes per each record. For prior HF, myocardial infarction, and other comorbidities, the codes in either the principal or the secondary diagnosis codes of the previous admissions up to 1 year before the index HF hospitalization were additionally identified to optimize the completeness of the data of comorbidities [11]. There were no changes in the ICD-10 TM code definitions during study period. The procedures performed during hospitalizations were identified by ICD-9 CM. The ICD-10 TM codes for comorbidities were grouped to be comparable to the Centers for Medicare and Medicaid Services ICD-10-CM mapping [11]. The full list of ICD-10 TM codes and ICD-9 CM codes used for grouping is provided in Additional file 1: Table S1.

### Primary and secondary outcomes

The main outcome was the annual rate of HF hospitalization and the trend from 2008 to 2013. To calculate the HF hospitalization rate per 100,000 beneficiaries for each year, we divided the number of hospitalized HF patient by the corresponding number of beneficiaries. The number of beneficiaries from the NHSO database were categorized by gender, age, and health security scheme. [12] The range of ages in the beneficiary data was categorized into 5-year intervals from 15–19 years, 20–24 years and etc., except for the oldest age category, ≥ 80 years. Thus, for the analysis of rate of HF hospitalization per 100,000 beneficiaries, we excluded patients aged 18–19 years from the analysis. Because one patient could have been admitted with HF more than once in each calendar year, we calculated HF hospitalization rate as unique person per 100,000 beneficiaries.

The in-hospital mortality rate was calculated by dividing the number of the hospitalizations ending in death identified by the discharge status code by the total of HF hospitalizations for a given year. The hospital length of stay (HLOS) was determined by the time and date of patient admission and discharge. A duration of stay > 6 h was counted as 1 day.

We calculated the all-cause HF rehospitalization and HF-specific rehospitalization rates. We identified rehospitalization after the index HF hospitalization using the encrypted national IDs. The time-to-rehospitalization was calculated by discharge date of the index HF hospitalization and the admission date of the subsequent hospitalization. The rehospitalization rate was calculated using the number of rehospitalizations in given period of interest (e.g., 30 days) divided by the number of index HF hospitalization discharged alive. Records were excluded from the rehospitalization analysis if the discharge status of the index HF hospitalization was either death or transferred. The cause of the rehospitalization was determined by the principal diagnosis of the readmission and was grouped by ICD-10 TM code. The ICD-TM code list for grouping the cause of rehospitalizations is shown in Additional file 1: Table S1.

The 1-year mortality rate was calculated by the time-to-death from the admission date of the index HF hospitalization to the date of death from the TCRD. If a patient as unique person was hospitalized for HF > 1 time in a given year, we randomly selected only one HF hospitalization in that calendar year [11]. We repeated this randomization of HF hospitalization selection three times to calculate the mortality rate. No significant differences in the 1-year mortality rate of each year was observed comparing between these three randomizations (data not shown).

### Statistical analysis

To test the statistical significance of changes over study period in patient characteristics, mortality rate, and rehospitalization rate, we used the Mantel–Haenszel-Chi-Squared test of linear association for categorical variables and the Cuzick non-parametric test for continuous variables [13].

For the annual rate of HF hospitalization per 100,000 beneficiaries, we evaluated significance of trend changes using Poisson regression. The age- and gender-adjusted HF hospitalization rate and incidence rate ratio compared to 2008 for each calendar year from 2009 to 2013 were calculated by Poisson regression. Unconditional binary logistic regression was used to test statistical significance of predictors for the outcomes of interest of rehospitalization and death.

Stata/MP, release 14.1 (StataCorp LP) was used for all analyses. A *p*-value < 0.05 was considered statistically significance using 2-sided tests.

## Results

**Fig. 1:**
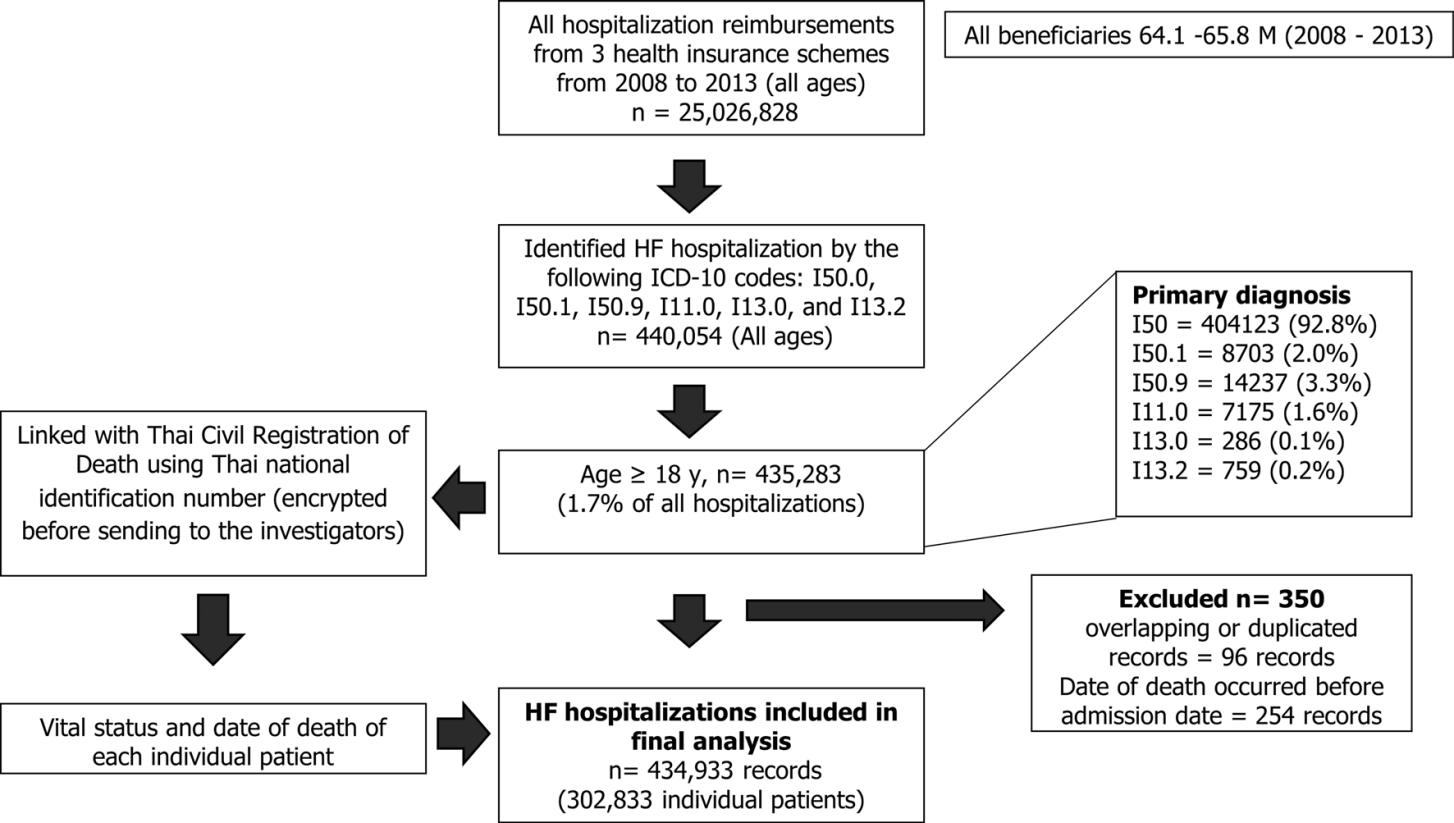
Flow chart of patient selection and distribution of ICD-10 codes of primary diagnosis. Abbreviations: *HF*, heart failure; *ICD-10*, International Classification of Diseases, Tenth Revision

From 2008 to 2013 nationwide hospital claims data, we identified 435,283 HF hospitalizations in patients aged ≥ 18 years, accounting for 1.7% of all the adult hospitalizations during the period. After cleaning the data, 434,933 HF hospitalizations in 302,833 patients were included in the final analysis. Figure 1 depicts a flow diagram of the patient selection and the distribution of the ICD-10 codes of the primary diagnosis. Most of the HF hospitalizations (92%) were under UCS coverage.

### Patient characteristics

Table 1 shows the characteristics of patients hospitalized for HF from 2008 to 2013. For the overall 6-year period, the mean age was 65.3 years (SD 14.6) without a significant linear trend over the study period (*P* for trend = 0.18). Those aged 60 to 79 years accounted for around half (51.4%) of all hospitalizations. The proportion of women hospitalized for HF (58.1%) was greater than that of men. Nearly half of the cohort (47.4%) had had a previous HF hospitalization during the previous year.

**Table 1:** Characteristics of hospitalized heart failure patients between 2008 and 2013 in Thailand

Parameter	Year						*P*-value for trend
	2008	2009	2010	2011	2012	2013	
HF hospitalizations, n	61,594	67,961	67,654	78,488	78,990	80,246	–
Age, mean (SD), y	64.4 (14.6)	64.6 (14.5)	66.0 (14.5)	65.2 (14.5)	64.9 (14.7)	65.2 (14.6)	0.180
*Age groups, n (%), years*							
18–39	3761 (6.1)	4024 (5.9)	3812 (5.6)	4355 (5.5)	4667 (5.9)	4460 (5.6)	0.004
40–59	17,121 (27.8)	18,737 (27.6)	18,309 (27.1)	20,885 (26.6)	21,384 (27.1)	21,437 (26.7)	0.001
60–79	31,958 (51.9)	35,284 (51.9)	35,179 (52.0)	40,798 (52.0)	40,246 (51.0)	41,101 (51.2)	0.019
≥ 80	8754 (14.2)	9916 (14.6)	10,354 (15.3)	12,450 (15.9)	12,693 (16.1)	13,248 (16.5)	< 0.001
Female	36,141 (58.7)	40,051 (58.9)	39,508 (58.4)	45,465 (57.9)	45,198 (57.2)	46,285 (57.7)	< 0.001
*Insurance scheme, n (%)*							
UCS	56,966 (92.5)	62,684 (92.2)	62,180 (91.9)	72,101 (91.9)	72,279 (91.5)	73,866 (92.0)	< 0.001
CSMBS	2281 (3.7)	2797 (4.1)	2721 (4.0)	3000 (3.8)	2825 (3.6)	2501 (3.1)	< 0.001
SSO	2347 (3.8)	2480 (3.6)	2753 (4.1)	3387 (4.3)	3886 (4.9)	3879 (4.8)	< 0.001
*Comorbidities, n (%)*^*a*^							
Prior HF	28,308 (46.0)	32,291 (47.5)	31,765 (47.0)	37,738 (48.1)	38,006 (48.1)	38,112 (47.5)	< 0.001
Prior MI	5881 (9.5)	6944 (10.2)	7188 (10.6)	8760 (11.2)	8986 (11.4)	8963 (11.2)	< 0.001
CAD	23,293 (37.8)	26,067 (38.4)	25,761 (38.1)	29,806 (38.0)	29,953 (37.9)	29,917 (37.3)	0.04
Non-rheumatic VHD	9452 (15.3)	10,862 (16.0)	10,649 (15.7)	12,641 (16.1)	12,732 (16.1)	13,343 (16.6)	< 0.001
Rheumatic VHD	6794 (11.0)	7413 (10.9)	7025 (10.4)	7989 (10.2)	7702 (9.8)	7696 (9.6)	< 0.001
Cardiomyopathy and myocarditis	4347 (7.1)	5090 (7.5)	5185 (7.7)	6418 (8.2)	6984 (8.8)	7557 (9.4)	< 0.001
Congenital heart disease	760 (1.2)	866 (1.3)	790 (1.2)	862 (1.1)	769 (1.0)	869 (1.1)	< 0.001
AF/AFL	13,830 (22.5)	15,818 (23.3)	15,902 (23.5)	19,127 (24.4)	19,079 (24.2)	19,944 (24.9)	< 0.001
Conduction abnormality	2441 (4.0)	2771 (4.1)	2799 (4.1)	3273 (4.2)	3366 (4.3)	3516 (4.4)	< 0.001
Ischemic stroke	2328 (3.8)	2670 (3.9)	2758 (4.1)	3439 (4.4)	3467 (4.4)	3567 (4.4)	< 0.001
Other CVDs	2042 (3.3)	2483 (3.7)	2496 (3.7)	3151 (4.0)	3251 (4.1)	3364 (4.2)	< 0.001
PAD and disease of aorta	359 (0.6)	411 (0.6)	417 (0.6)	462 (0.6)	509 (0.6)	484 (0.6)	< 0.001
Hypertension	32,707 (53.1)	38,359 (56.4)	39,945 (59.0)	48,294 (61.5)	50,382 (63.8)	52,692 (65.7)	< 0.001
Diabetes mellitus	20,282 (32.9)	23,172 (34.1)	23,826 (35.2)	27,886 (35.5)	28,609 (36.2)	30,018 (37.4)	< 0.001
Dyslipidemia	13,633 (22.1)	18,060 (26.6)	20,156 (29.8)	25,312 (32.2)	27,698 (35.1)	29,722 (37.2)	< 0.001
Renal failure	19,608 (31.8)	23,194 (34.1)	23,539 (34.8)	28,011 (35.7)	29,434 (37.3)	32,538 (40.5)	< 0.001
COPD	7163 (11.6)	8306 (12.2)	8274 (12.2)	9820 (12.6)	9929 (12.6)	9628 (12.0)	0.007
Liver disease	2993 (4.9)	3577 (5.3)	3506 (5.2)	4302 (5.5)	4544 (5.8)	4421 (5.5)	< 0.001
Depression	294 (0.5)	434 (0.6)	569 (0.8)	714 (0.9)	792 (1.0)	890 (1.1)	< 0.001
Dementia	120 (0.2)	207 (0.3)	188 (0.3)	234 (0.3)	228 (0.3)	254 (0.3)	0.001
HIV	389 (0.6)	424 (0.6)	433 (0.6)	478 (0.6)	572 (0.7)	559 (0.7)	0.015
Thalassemia	1004 (1.6)	1154 (1.7)	1192 (1.8)	1319 (1.7)	1422 (1.8)	1400 (1.7)	0.059
*Complications, n (%) *^*b*^							
VT/VF	204 (0.3)	224 (0.3)	285 (0.4)	314 (0.4)	288 (0.4)	330 (0.4)	0.015
Pneumonia	2961 (4.8)	3418 (5.0)	3686 (5.4)	4828 (6.2)	4915 (6.2)	5386 (6.7)	< 0.001
Acute kidney injury	2327 (3.8)	3045 (4.5)	3525 (5.2)	4488 (5.7)	5378 (6.8)	6305 (7.9)	< 0.001
Hyponatremia	3246 (5.3)	3926 (5.8)	4443 (6.6)	5991 (7.6)	5857 (7.4)	5915 (7.4)	< 0.001
Anemia	10,317 (16.8)	12,536 (18.4)	12,258 (18.6)	14,263 (18.2)	13,975 (17.7)	13,639 (17.0)	0.113

Abbreviations: AF/AFL, atrial fibrillation or atrial flutter; CAD, coronary artery disease; COPD, chronic obstructive pulmonary disease; CSMBS, Civil Servant Medical Benefit scheme; HIV, human immunodeficiency virus infection; HF, heart failure; CVD, cerebrovascular disease; MI, myocardial infarction; PAD, peripheral arterial disease; SSS, Social Security Scheme; UCS, Universal Health Coverage scheme; VHD, valvular heart disease; VT/VF, ventricular tachycardia or ventricular fibrillation

To test for significant trends, the Mantel–Haenszel-Chi-Square test of linear association for categorical variable and Cuzick non-parametric test for continuous variable were used

^a^ICD-10 coded in either index hospitalization or previous hospitalization claim record up to 1 year before index hospital

^b^ICD-10 coded in same hospitalization claim record

The most common comorbidity was hypertension (60.3%). About one-third of the patients had diabetes mellitus (35.4%), and dyslipidemia (30.1%). The proportion of coronary artery disease was 38.2%, and one-tenth (10.7%) had had a previous myocardial infarction. Codes defining atherosclerosis risk factors increased significantly over the study period (*P* for trend < 0.001 for each of hypertension, diabetes, and dyslipidemia). However, the proportion of coronary artery disease as a comorbidity was relatively unchanged. The proportion of rheumatic valvular heart disease as a comorbidity decreased, but those of non-rheumatic valvular heart diseases, cardiomyopathies, and atrial fibrillation increased. The proportions of several other comorbidities and complications, including renal failure, anemia, and pneumonia increased.

### HF hospitalization rate per 100,000 beneficiaries

**Fig. 2:**
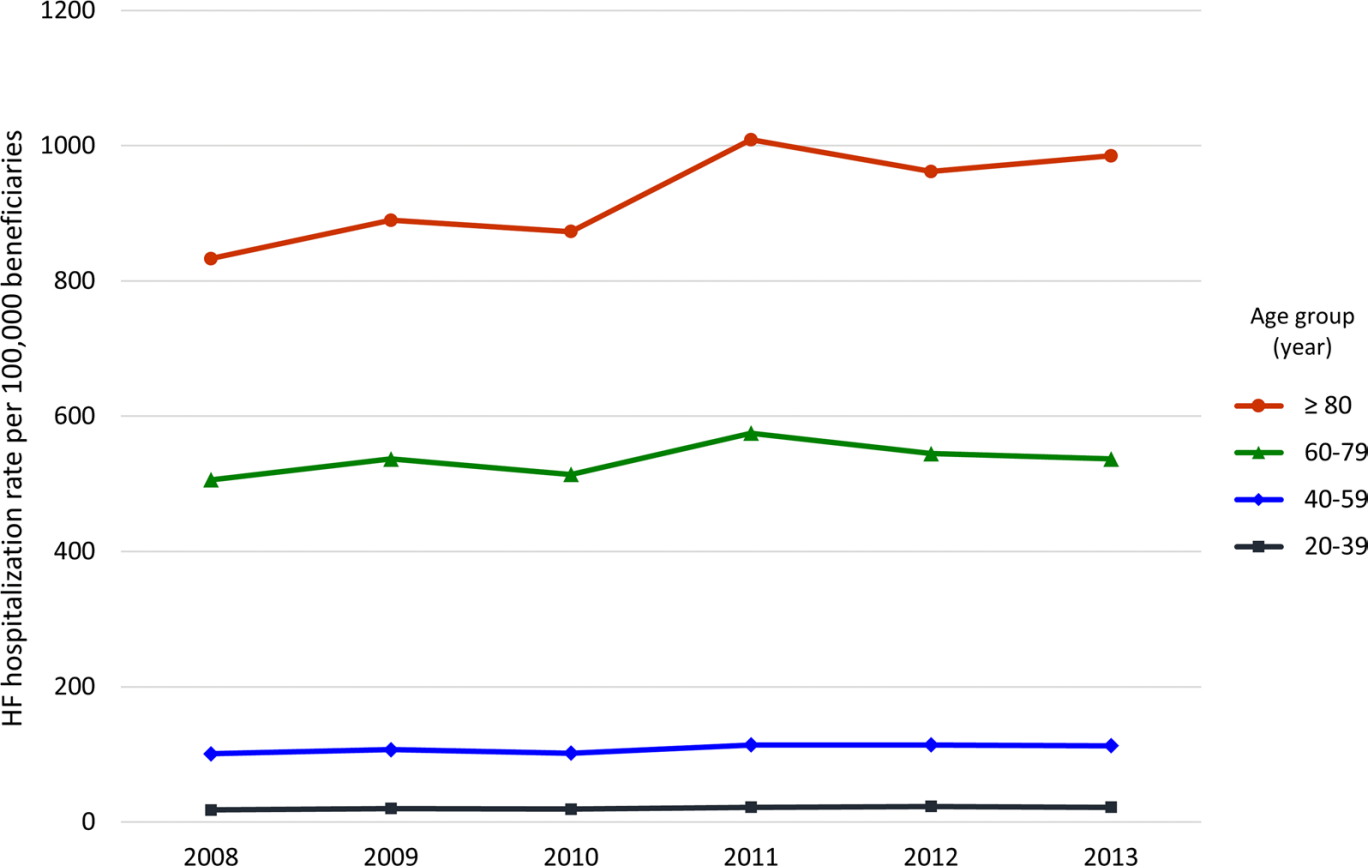
Heart failure hospitalization rate per 100,000 beneficiaries stratified by age group between 2008 and 2013. All age groups had significantly upward trend for heart failure (HF) hospitalization rate per 100,000 beneficiaries. *P*-values for trend were < 0.001 for all age groups using linear trend across years by Poisson regression

Table 2 shows the numbers and annual rates of HF hospitalization from 2008 to 2013. Between 2008 and 2013, there was an increase in the number of annual hospitalizations from 61,594 to 80,246, respectively (an increase of 30.3%), number of patients from 43,326 to 55,864 per year, respectively (an increase of 28.9%), and the rate of HF hospitalization from 138 to 168 per 100,000 beneficiaries, respectively (an increase of 21.7%) (all *P* for trend < 0.001). After adjusting for age and sex, the trend of HF hospitalization rate was still significant (*P* for trend < 0.001). The increasing HF hospitalization rate was driven mostly by the patients under UCS. The oldest age group (≥ 80 years) had the highest HF hospitalization rate of 931 per 100,000 beneficiaries for the overall period. This group also had the most prominent upward trend in the number of hospitalizations, increasing from 8754 to 13,248 from 2008 to 2013 (an increase of 51.3%) (*P* for trend < 0.001 in Table 1). All age groups had significant upward trends in HF hospitalization rates, especially in age ≥ 80 group as shown in Fig. 2 (*P* for trend < 0.001 for all age groups). Hospitalization for HF was more common in females (188 per 100,000 female beneficiaries vs. 147 per 100,000 male beneficiaries) (Table 2), especially in those aged ≥ 60 years, and the HF hospitalization rates for both genders significantly increased (both *P* < 0.001).

**Table 2:** Heart failure hospitalization rate per 100,000 beneficiaries in Thailand between 2008 and 2013^a^

Parameter	Year						*P*-value for trend ^b^
	2008	2009	2010	2011	2012	2013	
Beneficiaries, n of persons	44,448,763	44,954,097	45,539,165	46,352,534	47,184,394	47,643,858	–
HF hospitalization, n	61,472	67,833	67,545	78,368	78,849	80,096	< 0.001
HF hospitalization rate, per 100,000 beneficiaries	138	151	148	169	167	168	< 0.001
Unique HF patients, n	43,236	46,967	47,495	54,075	54,745	55,762	–
HF hospitalization rate of unique HF patients, per 100,000 beneficiaries	97	104	104	117	116	117	< 0.001
*HF hospitalization rate per 100,000 beneficiaries stratified by specified groups*							
By insurance scheme							
UCS	184	198	195	223	219	223	< 0.001
CSMBS	57	70	68	73	68	61	0.101
SSS	24	26	28	34	38	37	< 0.001
By age group, years							
20–39	18	20	19	22	23	22	< 0.001
40–59	101	107	102	114	114	113	< 0.001
60–79	506	537	514	575	545	537	< 0.001
≥ 80	833	890	873	1009	962	985	< 0.001
By gender							
Male	118	128	128	147	148	147	< 0.001
Female	157	172	168	190	185	188	< 0.001
Age and gender-adjusted HF hospitalization rate, per 100,000 beneficiaries (95% CI) ^c^	138 (138–138)	147 (146–149)	142 (140–143)	159 (157–161)	154 (152–156)	153 (151–154)	< 0.001
Incidence rate ratio (95% CI) ^c^	Ref	1.07 (1.05–1.08)	1.02 (1.01–1.03)	1.15 (1.14–1.16)	1.11 (1.10–1.13)	1.1 (1.09–1.12)	< 0.001

Abbreviations: *CI*, confidence interval; *CSMBS*, Civil Servant Medical Benefit scheme; *HF*, heart failure; *ref*., reference category; *SSS*, Social Security scheme; *UCS*, Universal Health Coverage scheme

^a^Only beneficiaries aged ≥ 20 years were analyzed

^b^Linear trend across years by Poisson regression

^c^Age and gender-adjusted incidence rate ratio compared to 2008 were calculated by Poisson regression for each year from 2009 to 2013

### In-hospital procedures and outcomes

The median HLOS was 3 days (IQR 1.9–5.0) (*P* for trend = 0*.*42) (Table 3). There were increasing trends in some procedural codes, including mechanical ventilator from 6.7 to 8.9% and echocardiography from 4.9 to 9.0% (both *P* for trend < 0.001). In-hospital mortality rate gradually declined from 4.4% in 2008 to 3.8% in 2013 (*P* for trend < 0.001) with modest change after adjusting for age and sex (*P* for trend < 0.001). The significant predictors of in-hospital mortality were the extreme age groups, (i.e., the youngest age category and the oldest age category), concomitant cerebrovascular disease, renal and liver failure, and the presence of codes for complications or organ support procedures, such as mechanical ventilator (Additional file 1: Table S2).

**Table 3:** In-hospital procedures and outcomes associated with heart failure hospitalization in Thailand between 2008 and 2013

Parameter	Year						*P-*value for trend^a^
	2008	2009	2010	2011	2012	2013	
HF hospitalization, n	61,594	67,961	67,654	78,488	78,990	80,246	–
Hospital length of stay, median (IQR), d	3.0 (1.9–5.0)	3.0 (1.9–5.0)	3.0 (1.9–5.0)	3.0 (1.9–5.0)	3.0 (1.9–5.0)	3.0 (1.9–5.0)	0.42
In-hospital mortality, n (%)	2690 (4.4)	2980 (4.4)	2912 (4.3)	3126 (4.0)	3153 (4.0)	3061 (3.8)	< 0.001
Age and gender-adjusted in-hospital mortality rate, %^b^	4.4	4.3	4.3	4.3	4.3	4.1	< 0.001
*Procedure, n (%)*^*c*^							
Ventilator	4141 (6.7)	4939 (7.3)	5391 (8.0)	6552 (8.3)	6648 (8.4)	7105 (8.9)	< 0.001
Echocardiography	3037 (4.9)	4367 (6.4)	4987 (7.4)	6116 (7.8)	6544 (8.3)	7218 (9.0)	< 0.001
Cardiac catheterization	273 (0.4)	331 (0.5)	384 (0.6)	391 (0.5)	571 (0.7)	764 (1.0)	< 0.001
Percutaneous coronary intervention	26 (0.1)	40 (0.1)	41 (0.1)	42 (0.1)	54 (0.1)	81 (0.1)	0.803
Renal replacement therapy	350 (0.6)	518 (0.8)	559 (0.8)	547 (0.7)	623 (0.8)	638 (0.8)	< 0.001
Cardioversion / defibrillation	96 (0.2)	161 (0.2)	190 (0.3)	231 (0.3)	240 (0.3)	243 (0.3)	< 0.001
Cardiopulmonary resuscitation	744 (1.2)	945 (1.4)	953 (1.4)	1096 (1.4)	1057 (1.3)	1035 (1.3)	0.711

Abbreviations: HF, heart failure; IQR, interquartile range

^a^To test for significant trends, the Mantel–Haenszel-Chi-Square test of linear association for categorical variables and Cuzick non-parametric test for continuous variable were used

^b^Age and gender at 2008 were used to adjusted using Poisson regression

^c^Procedure coded by ICD-9

### Rehospitalization rate

Overall, rehospitalization rates were 34% in 30 days and 73% in 1 year after discharge without a significant linear trend over study period (*P* = 0.186 and 0.084 for 1-month and 1-year rehospitalization rate, respectively) (Table 4). The median time for all-cause hospitalization after the index HF hospitalization was 37 days (IQR 12–105).

**Table 4:** Rehospitalization rate and 1-year mortality rate in Thailand between 2008 and 2013^a^

Parameter	Year						*P*-value for trend
	2008	2009	2010	2011	2012	2013^b^	
HF hospitalization, n	61,594	67,961	67,654	78,488	78,990	80,246	–
Discharged alive HF hospitalization, n	54,280	59,964	59,629	69,343	70,143	64,672	–
30-Day all-cause rehospitalization rate, n (%)	18,471 (34.0)	20,640 (34.4)	20,458 (34.3)	23,801 (34.3)	24,184 (34.5)	22,386 (34.6)	0.186
Age and sex-adjusted 30-day rehospitalization rate, %^d^	33.9	34.3	34.3	34.4	34.5	34.7	< 0.001
One-year rehospitalization	39,254 (72.3)	43,662 (72.8)	43,883 (73.6)	50,776 (73.2)	51,486 (73.4)	NA	0.084
Time to first all-cause rehospitalization, median (IQR), d	37 (12–105)	36 (12–104)	37 (13–106)	37 (12–105)	37 (12–104)	NA	0.371
*HF rehospitalization*							
30-Day HF rehospitalization, n (%)	8663 (16.0)	9840 (16.4)	9602 (16.1)	11,484 (16.6)	11,668 (16.6)	10,799 (16.7)	0.001
Age–sex-adjusted 30-day HF rehospitalization rate, %	16.0	16.4	16.1	16.6	16.6	16.7	0.001
Three-month HF rehospitalization	14,707 (27.1)	16,637 (27.7)	16,362 (27.4)	19,281 (27.8)	19,508 (27.8)	NA	0.054
One-year HF rehospitalization	22,134 (40.8)	24,691 (41.2)	24,737 (41.5)	28,708 (41.4)	28,773 (41.0)	NA	0.543
Time for first HF rehospitalization, median (IQR), d	49 (16–128)	48 (16–125)	49 (17–131)	48 (16–126)	47 (16–124)	NA	0.063
*One-year mortality*							
HF patients, n of persons	43,323	47,060	47,584	54,156	54,835	NA	
One-year mortality	13,766 (31.8)	14,440 (30.7)	14,347 (30.2)	16,072 (29.7)	15,641 (28.5)	NA	< 0.001
Age and sex-adjusted 1-year mortality, % (95% CI)^c^	31.9 (31.5–32.4)	30.8 (30.4–31.2)	30.1 (29.7–30.5)	29.6 (29.2–30.0)	28.4 (28.1–28.8)	NA	NA

Abbreviations: *CI*, confidence interval; *HF*, heart failure; *IQR*, interquartile range; *NA*, not applicable

^a^To test for significant trends, the Mantel–Haenszel-Chi-Square test of linear association for categorical variables and Cuzick non-parametric test for continuous variable were used

^b^Excluded patient discharged in December 2013 for 30-day rehospitalization rate

^c^Age and gender at 2008 were used to adjusted using Poisson regression

The most common cause for 30-day rehospitalization was HF, accounting for 42% of all the rehospitalizations. Other common coding groups for 30-day rehospitalization were coronary artery disease (9%) and other cardiovascular condition (10%). Non-cardiovascular conditions contributed to 39% of all the rehospitalization. The most common non-cardiovascular condition causing rehospitalization was renal failure (7%).

The 30-day HF rehospitalization rate was around 16% with a slight increase over the study period (*P* for trend = 0.001). The trend persisted after adjusting for sex and age (*P* for trend = 0.001). The 3-month and 1-year HF rehospitalization rates were 27 and 41%, respectively, without a significant linear trend. Most of the HF rehospitalizations occurred at a median time-after-discharge of 48 days (IQR 16–126).

The factors associated with 30-day HF rehospitalization are reported in Additional file 1: Table S3. The youngest age group was more likely to have subsequent HF rehospitalization (20.7 vs. 16.4% overall). Prior HF admission indicated greater risk for future HF rehospitalization (OR 3.35, 95% CI 3.29–3.42). Various cardiovascular and other comorbidities increased the chance of HF readmission such as previous MI (OR 1.92, 95% CI 1.87–1.96). Admission records coded with an echocardiography procedure had a significantly lower rate of 30-day HF rehospitalization (OR 0.65, 95% CI 0.63–0.68).

### One-year mortality rate

One-year mortality rate significantly decreased from 31.8 in 2008 to 28.5% in 2012 (both unadjusted and age- and sex-adjusted *P* for trend < 0.001) (Table 4). Additional file 1: Table S4 shows the predictors of 1-year mortality. The oldest age group had nearly twice the risk of 1-year mortality (OR 1.93, 95% CI 1.85–2.01). Male had a slightly but significantly greater risk than female (OR 1.13, 95% CI 1.11–1.15). Dissimilar to in-hospital mortality, previous hospitalization for HF or myocardial infarction markedly increased the risk of 1-year mortality (OR 1.48, 95% CI 1.46–1.51 and OR 1.45, 95% CI 1.41–1.50, respectively). The mortality risk was lower for patients undergoing echocardiography (OR 0.75, 95% CI 0.73–0.78) and cardiac catheterization codes (OR 0.44, 95% CI 0.38–0.51).

## Discussion

To the best of our knowledge, this is the first national population study of hospitalized HF patients in Thailand. Using routinely-collected claims data from three public health security schemes, we found increasing trends in both the number and HF hospitalization rate between 2008 and 2013, driven mostly by the elderly population. The HF hospitalization rate increased with age and was higher in female. The in-hospital and 1-year mortality rates improved slightly between 2008 and 2013. Nevertheless, one-third of these patient were rehospitalized within 1 month, and nearly half of these rehospitalizations were caused by recurrent HF.

The increasing HF hospitalization rate in Thailand is probably caused by a growing number of HF patients in Thai population. The number of elderly and the proportion of the older population in Thailand have been increasing rapidly [14]. HF is more common with increasing age; the elderly population has a higher prevalence of cardiovascular disease, particularly hypertension, valvular heart disease, and coronary heart disease, which can lead to HF. Aging by itself is also an important risk factor for developing HF [15]. Moreover, a sedentary lifestyle with insufficient physical activity and dietary change to a Western diet might lead to a greater prevalence of HF risk factors, such as obesity, diabetes mellitus, and hypertension [16, 17]. Conversely, improved public health care provisions might also contribute to an increase in access to health care services, including hospitalization. Evidenced by the same set of claims data, we observed an increase of 14.2% in any hospitalizations from 5,041,334 in 2008 to 5,757,022 admissions in 2013. However, more access to health care services in high-income countries (HICs) led to fewer hospitalizations due to chronic disease, including HF [18].

Epidemiologic data on HF in LMICs are scarce and are often based on patients hospitalized for acute HF. The true prevalence of HF patient and trends in this region remain unknown [16]. Between HICs, HF hospitalization rates and trends are discrepant. For example, decreasing HF hospitalization rates per population were observed in the United States of America (USA), England, France, and Ireland over a similar time period to the present study [19–22]. However, the crude HF hospitalization rates were increasing in Germany and Slovenia [23, 24] The differences in rates and trends between countries might be partially explained by heterogenous research methods to identify HF patients, healthcare provision accessibility, and dissimilarity in medical management. In chronic HF, guideline-directed medical therapy can substantially reduce hospitalization events and mortality rate, especially in those with reduced left ventricular ejection fraction [25]. However, these life-saving therapies are still underused even in HICs [26]. Health care policy and quality measurement might also affect the HF hospitalization rate [27].

The characteristics of HF patients in the present study are partly consistent with the Thai ADHERE study and ADHERE-Asia–Pacific study [5, 6], which demonstrated younger patient (mean age of 65 years compared to 72 years in USA [19] and reaching 80 years in some European countries) [21, 24]. Female was more prevalent (58%) in the present study with a higher HF hospitalization rate per population, especially in the older age group. However, other population-based studies in the USA and Singapore showed male had a comparable hospitalization rate [19, 28] or even a higher rate than that of female in France [21]. In the present study, the reasons for the higher HF hospitalization rate in women were not explored, but it was possibly due to the higher prevalence of diabetes and obesity in Thai women, which are risk factors for developing HF [17, 29]. About one-tenth of the hospitalizations had rheumatic heart disease as a comorbidity with a downward trend. In addition, hypertension, diabetes mellitus, and coronary artery disease showed an increasing trend over the study period. Therefore, these metabolic disorders increasingly contribute to HF similar to Western countries [30].

In the present study, the in-hospital mortality rate slightly declined over the study period. Despite of lower mean age, the in-hospital mortality rate was higher than that of a cohort from the United States (4.1 vs. 3.0%) [5]. In the present study, we found a higher rate of mechanical ventilator use (up to 8.9%), indicating a greater severity of HF, especially in the youngest group (age 18–40 years), which had the highest mortality rate of 5.3%. However, some studies from HICs in Europe reported higher mortality rates (6.7–9.3%) and longer HLOS [21, 23, 31] This was probably due to older HF patients. Cohorts of hospitalized HF in high-income Asian countries such as Japan and Korea also demonstrated longer HLOS and high mortality of 6.4 and 5.2%, respectively [32, 33]. Therefore, the lower mortality rate in the cohort from the United States might be partially explained by a much higher HF hospitalization rate in the USA, which probably included less severe patient and shorter HLOS, which might have resulted in post-discharge mortality or readmission instead [34].

In the present study, echocardiography was coded more frequently between 2008 and 2013, reaching 9.0% in 2013. However, this is a small proportion compared with 62–85% performed in Europe and the USA [5]. Echocardiography is the most useful single test for evaluating the cause of HF and guiding the recommended treatment [35]. Moreover, patients who underwent echocardiography during admission had a better 1-year survival rate and less HF hospitalization in the present study. Sonographer-driven echocardiographic study with web-based assessment is feasible and might be applied to hospitals in remote areas with limited cardiology services [36].

Compared to other conditions, patients hospitalized with HF have the highest rate of rehospitalization [1]. In the present study, the 30-day rehospitalization rate was 34%, which is higher than in HICs countries in which the rate was lower than one-third [21, 24, 37–39]. The most common cause of rehospitalization in the present study was the decompensation of HF, found in nearly half of the rehospitalizations, and this is a higher proportion compared to the one-third of rehospitalization found in other studies [1, 40]. However, the risk of HF rehospitalization is modifiable, and there is much room for improvement for HF care in Thailand. For example, neurohormonal blocker was prescribed in less than half of the hospitalized HF patients in the Thai ADHERE study [6]. The possible solutions are to optimize HF management and echocardiographic evaluation before discharge. Early follow-up visits for high-risk patients and implementation of guideline directed treatment with comprehensive management can be achieved by a multidisciplinary team [41, 42]. Appropriate health care policy and strategy are needed to implement these into real-world practice [37, 43].

In the present study, 1-year mortality rate improved over the study period and was 28.5% in 2013. However, this is still substantially higher than in HICs, which ranged from 8.9 to 23.6% [44–47]. This is consistent with International Congestive Heart Failure Cohort (INTER-CHF), which showed high variation in mortality rates in LMICs, including Southeast Asian countries [48]. The INTER-CHF also demonstrated the differences in HF medication and socioeconomic status affecting the outcome of HF patients. Other unmeasured factors, including health-care quality and accessibility along with environmental and genetic factors, might also play roles [48]. Moreover, implantable cardioverter defibrillator (ICD), which prevents sudden cardiac death in HF, is also underutilized in lower income Asian countries. Only 15% of eligible HF patients had an ICD implanted in Thailand according to the Asian Sudden Cardiac Death in Heart Failure (ASIAN-HF) registry [49].

## Limitations

Several limitations of our study warrant discussion. First, the claims data did not include HF hospitalizations in some private hospitals because they do not participate in the public health security reimbursement program, which is mainly UCS. People with high social economic status or private health insurance might choose to be hospitalized in private hospitals even though they have public health protection coverage. Therefore, our analysis likely underestimates the national HF hospitalization rate. The extent of the lack of coverage of private hospitals in our data collection is difficult to estimate. Additionally, the outcomes of the patients hospitalized for HF in private hospitals not participating in the public health security program might be different from the present study possibly due to higher socioeconomic status or quality of care [50, 51]. Second, we identified the HF hospitalizations based on ICD codes without validation using clinical data. Despite auditing by the BCMA, coding misclassification or changing coding practices are possible. There is no existing validation study on HF hospitalization based on hospital discharge data in Thailand. Moreover, HF diagnosis may have been misclassified due to lack of expertise or resource limitation, especially in rural hospitals. These possibilities might be demonstrated by the low coding rate for echocardiography in the present study. Although, heart failure is a clinical syndrome, lack of echocardiographic study likely reduced the diagnostic accuracy for this condition. Third, the rehospitalization rate is probably underestimated because patients might have decided to receive treatment in a private hospital not covered by our data collection after the index HF hospitalization, thereby not being included in our data source. Fourth, there are insufficient data for the cause of death analysis mainly due to the non-specific causes of death given in death certificate data. Fifth, interpreting factors associated with the outcomes in the present study should be done with caution because these variables were determined by administrative database data. Furthermore, without the availability of clinical data to adjust for more confounders, there might be residual confounding of estimated associations, and we did not perform multivariable analysis given these limitations. Sixth, our HF hospitalization data do not represent the epidemiology of chronic HF, which should have a higher incidence. However, HF hospitalization rate can still reflect health and economic burden of this condition. Finally, our data from 2013 are considered quite old. Due to lengthy process in requesting the claim data and data linkage which involve many government offices, obtaining new dataset will take considerable amount of time. Moreover, the patient ID were encrypted, so combining the old requested data with the new data will be very complex task. Nevertheless, our study, as the first nationwide HF study in Thailand, would be an important reference to compare HF epidemiology and burden in Thailand and other low-to-medium income country in the future study.

## Future perspectives

We are facing a HF epidemic, especially in LMICs, including Thailand. Optimizing modifiable cardiovascular risk factor control, particularly hypertension, is necessary to reduce HF hospitalizations [52]. However, there are the difference in the comorbidities and characteristics of HF patients between the Asian region and Western countries [30]. Prospective population-based studies of HF epidemiology are needed to understand the etiology and prognostic factors of HF in this region. Establishing registries and performing registry-based studies to evaluate the quality of evidenced-based HF treatment might help address the gaps and identify the opportunities to improve patient care for HF with reduced ejection fraction [26]. Nurse-led HF clinics with established protocols can reduce HF hospitalizations and mortality, especially in setting where the physician’s time is limited.^53^ Implementation of this kind of clinic also has the potential to create a network for patient transfers and the platform for research registries in the future.

## Conclusion

In this national population study, there was a trend of increasing HF-hospitalized patients in Thailand from 2008 to 2013. The risk of HF hospitalization increased with age, and a greater proportion were female. In-hospital and 1-year mortality rates gradually improved during the study period. However, the 30-day HF rehospitalization rate remained high.

## Supplementary Information

**Additional file 1.** Table S1 (list of ICD code for grouping comorbidities and procedure) and Table S2 - S4 (Parameters associated with in-hospital mortality, 30-day rehospitalization and 1-year mortality).

## Data Availability

The datasets used in the current study are available from the corresponding author upon reasonable request.

## Abbreviations

CSMBS: Civil Servant Medical Benefit scheme
HIC: High-income county
HF: Heart failure
HLOS: Hospital length of stay
ICD: Implantable cardioverter defibrillator
ICD-9 CM: International Classification of Diseases, Ninth Revision, Clinical Modification
ICD-10 TM: The International Classification of Diseases, Tenth Revision, Thai modification
LMIC: Low- and middle- income country
SSS: Social Security Scheme
TRCD: Thai Civil Registration of Death
UCS: Universal Health Coverage scheme
