# Supplementary material for: National trend in heart failure hospitalization and outcome under public health insurance system in Thailand 2008–2013
Source: BMC Cardiovasc Disord. 2022 Apr 29;22:203. doi: 10.1186/s12872-022-02629-2 (PMC9052701; doi:10.1186/s12872-022-02629-2)
Supplement: Supplementary file 1 — Additional file 1. Table S1 (list of ICD code for grouping comorbidities and procedure) and Table S2 - S4 (Parameters associated with in-hospital mortality, 30-day rehospitalization and 1-year mortality). [file 12872_2022_2629_MOESM1_ESM.docx]

# **Table S1**. List of ICD-9 and ICD-10 code for grouping comorbidities and procedures

| Number |  | **ICD 10 / ICD 9** |
| --- | --- | --- |
| **Comorbidities as underlying disease: ICD-10 in secondary diagnoses in same visit or**  **in either the principle or secondary diagnoses in the previous admission within 1 year** | | |
| 1 | Prior heart failure | I110,I130,I132,I500,I501,I509 |
| 2 | Prior myocardial infarction | I21,I210,I211,I212,I213,I214,I219,I22,I220,I221,I228,I229 |
| 3 | Coronary artery disease, excluding I23 complication of myocardial infarction, I24 other | I20,I200,I201,I208,I209,I21,I210,I211,I212,I213,I214,I219,I22,I220,I221,I228,I229,I25,I250,I251,I252,I253,I254,I255,I256,I258,I259 |
| 4 | non-rheumatic valvular heart disease (I34-I39) , I511 (rupture chordae) | I34,I340,I341,I342,I348,I349,I35,I350,I351,I352,I358,I359,I36,I360,I361,I362,I368,I369,I37,I370,I371,I372,I378,I379,I38,I39,I390,I391,I392,I393,I394,I398,I511 |
| 5 | Rheumatic valvular heart disease (I05-I09) | I05,I050,I051,I052,I058,I059,I06,I060,I061,I062,I068,I069,I07,I070,I071,I072,I078,I079,I08,I080,I081,I082,I083,I088,I089,I09,I090,I091,I092,I098,I099 |
| 6 | Cardiomyopathy and myocarditis (I40-I43) | I40,I400,I401,I408,I409,I41,I410,I411,I412,I418,I42,I420,I421,I422,I423,I424,I425,I426,I427,I428,I429,I43,I430,I431,I432,I438 |
| 7 | Congenital heart disease (Q20-Q264), including Marfan syndrome (Q874) | Q20,Q200,Q201,Q202,Q203,Q204,Q205,Q206,Q208,Q209,Q21,Q210,Q211,Q212,Q213,Q214,Q218,Q219,Q22,Q220,Q221,Q222,Q223,Q224,Q225,Q226,Q228,Q229,Q23,Q230,Q231,Q232,Q233,Q234,Q238,Q239,Q24,Q240,Q241,Q242,Q243,Q244,Q245,Q246,Q248,Q249,Q25,Q250,Q251,Q252,Q253,Q254,Q255,Q256,Q257,Q258,Q259,Q26,Q260,Q261,Q262,Q263,Q264,Q874 |
| 8 | Atrial fibrillation / atrial flutter | I48 |
| 9 | Conduction abnormality, including sick sinus syndrome and bradycardia (R001) | I44,I440,I441,I442,I443,I444,I445,I446,I447,I45,I450,I451,I452,I453,I454,I455,I456,I458,I459,I495,R001 |
| 10 | Ischemic stroke and sequelae (I693), including transient ischemic attack (G45x) and vascular syndrome (G46), Z867 (old cerebrovascular disease with full recovery) excluding I65-I66 (does not result in stroke) | I63,I630,I631,I632,I633,I634,I635,I636,I638,I639,I64,I693,G45,G450,G451,G452,G453,G454,G458,G459,G46,G460,G461,G462,G463,G464,G465,G466,G467,G468,Z867 |
| 11 | Other cerebrovascular disease (I60 – I 69), excluding I63-I66x and sequelae | I60,I600,I601,I602,I603,I604,I605,I606,I607,I608,I609,I61,I610,I611,I612,I613,I614,I615,I616,I618,I619,I62,I620,I621,I629,I670,I671,I672,I673,I674,I675,I676,I677,I678,I679,I68,I680,I681,I682,I688,I69,I690,I691,I692,I694,I698 |
| 12 | Peripheral arterial disease and disease of aorta (including dissection and aneurysm) | I70,I700,I7000,I7001,I701,I7010,I7011,I702,I7020,I7021,I7022,I7029,I703,I708,I7080I7081,I709,I7090,I7091,I71,I710,I711,I712,I713,I714,I715,I716,I718,I719,I72,I720,I721,I722,I723,I724,I725,I728,I729 |
| 13 | Hypertension | I10,I15,I150,I151,I152,I158,I159 |
| 14 | Diabetic mellitus | E10,E100,E101,E102,E103,E104,E105,E106,E107,E108,E109,E11,E110,E111,E112,E113,E114,E115,E116,E117,E118,E119,E12,E120,E121,E122,E123,E124,E125,E126,E127,E128,E129,E13,E130,E131,E132,E133,E134,E135,E136,E137,E138,E139,E14,E140,E141,E142,E143,E144,E145,E146,E147,E148,E149 |
| 15 | Dyslipidemia | E78,E780,E781,E782,E783,E784,E785,E786,E788,E789 |
| 16 | Renal failure | R392,N17,N170,N171N172,N178,N179,N18,N180,N181,N182,N183,N184,N185,N188,N189,N19 |
| 17 | Chronic obstructive pulmonary disease | J41,J410,J411,J418,J42,J43,J430,J431,J432,J438,J439,J44,J440,J441,J442,J448,J449 |
| 18 | Liver disease | K70,K700,K701,K702,K703,K704,K709,K71,K710,K711,K712,K713,K714,K715,K716,K717,K718,K719,K72,K720,K721,K729,K73,K730,K731,K732,K738,K739,K74,K740,K741,K742,K743,K744,K745,K746,K75,K750,K751,K752,K753,K754,K758,K759,K76,K760,K761,K762,K763,K764,K765,K766,K767,K768,K769,K77,K770,K778 |
| 19 | Depression | F32,F320,F3200,F3201,F321,F3210,F3211,F322,F323,F3230,F3231,F328,F329,F33,F330,F3300,F3301,F331,F3310,F3311,F332,F333,F3330,F3331,F334,F338,F339 |
| 20 | Dementia (F00-F03) | F00,F000,F0000,F0001,F0002,F0003,F0004,F001,F0010,F0011,F0012,F0013,F0014,F002,F0020,F0021,F0022,F0023,F0024,F009,F0090,F0091,F0092,F0093,F0094,F01,F010,F0100,F0101,F0102,F0103,F0104,F011,F0110,F0111,F0112,F0113,F0114,F012,F0120,F0121,F0122,F0123,F0124,F013,F0130,F0131,F0132,F0133,F0134,F018,F0180,F0181,F0182,F0183,F0184,F019,F0190,F0191,F0192,F0193,F0194,F02,F020,F0200,F0201,F0202,F0203,F0204,F021,F0210,F0211,F0212,F0213,F0214,F022,F0220,F0221,F0222,F0223,F0224,F023,F0230,F0231,F0232,F0233,F0234,F024,F0240,F0241,F0242,F0243,F0244,F028,F0280,F0281,F0282,F0283,F0284,F03,F030,F031,F032,F033,F034 |
| 21 | Human immunodeficiency virus infection include R75 (positive test) and Z21(asymptomatic) | B20,B200,B201,B202,B203,B204,B205,B206,B207,B208,B209,B21,B210,B211,B212,B213,B217,B218,B219,B22,B220,B221,B222,B227,B23,B230,B231,B232,B233,B238,B24,R75,Z21 |
| 22 | Thalassemia | D56,D560,D561,D562,D563,D564,D568,D569 |
| **Complications: ICD 10 - ONLY secondary diagnoses in the same visit** | | |
| 23 | Ventricular tachycardia and ventricular fibrillation | I470,I472,I490,I4900,I4901,I4908,I4909 |
| 24 | Pneumonia include J851 (lung abscess) | J100,J110,J12,J120,J121,J122,J123,J128,J129,J13,J14,J15,J150,J151,J152,J153,J154,J155,J156,J157,J158,J1581,J1588,J159,J16,J160,J168,J17,J170,J171,J172,J173,J178,J18,J180,J181,J182,J188,J189,J851 |
| 25 | Acute renal failure | R392,N17,N170,N171N172,N178,N179 |
| 26 | Hyponatremia | E871 |
| 27 | Anemia | D50,D500,D501,D508,D509,D51,D510,D511,D512,D513,D518,D519,D52,D520,D521,D528,D529,D53,D530,D531,D532,D538,D539,D55,D550,D551,D552,D553,D558,D559,D56,D560,D561,D562,D563,D564,D568,D569,D57,D570,D571,D572,D573,D578,D58,D580,D581,D582,D588,D589,D59,D590,D591,D592,D593,D594,D5940,D5941,D5942,D5943,D5948,D595,D596,D598,D599,D60,D600,D601,D608,D609,D61,D610,D611,D612,D613,D618,D619,D62,D63,D630,D631,D638,D64,D640,D641,D642,D643,D644,D648,D649, |
| **Procedure: ICD 9 in index heart failure hospitalization** | | |
| 28 | Ventilator: any duration | 967,9670,9671,9672 |
| 29 | Need for ventilator < 96 h | 9671 |
| 30 | Need for ventilator > 96 h | 9672 |
| 31 | Echocardiography | 8872 |
| 32 | Cardiac catheterization, coronary angiography, exclude 8851 (Inferior vena cava angiography) | 3721,3722,3723,885,8850,8852,8853,8854,8855,8856,8857 |
| 33 | Percutaneous coronary intervention | 0066,3606,3607,3609 |
| 34 | Implantable cardioverter defibrillator | 0051,3794 |
| 35 | Cardiac resynchronization therapy | 0050,0051 exclude replace lead/pulse generator only (0053,0054,3795,3796,379 7,3798) |
| 36 | Heart transplantation | 3751, exclude heart-lung transplantation (336) |
| 37 | Renal replacement therapy | 3995,5498 |
| 38 | Cardioversion and defibrillation | 9961,9962 |
| 39 | Cardiopulmonary defibrillation | 9960, 9963 |
| **Grouping cause of rehospitalization: principal diagnosis in ICD-10 code after index hospitalization** | | |
| 40 | Heart failure | I50,I500,I501,I509,I110,I130,I132 |
| 41 | Coronary artery disease | I20,I200,I201,I208,I209,I21,I210,I211,I212,I213,I214,I219,I22,I220,I221,I228,I229,I23,I230,I231,I232,I233,I234,I235,I236,I238,I24,I240,I241,I248,I249,I25,I250,I251,I252,I253,I254,I255,I256,I258,I259, |
| 42 | Renal failure | R392.N17.N170.N171.N172.N178.N179.N18.N180.N181.N182.N183.N184.N185.N188.N189.N19.N10. |
| 43 | Respiratory tract infection | J100,J101,J108,J11,J110,J111,J118,J12,J120,J121,J122,J123,J128,J129,J13,J14,J15,J150,J151,J152,J153,J154,J155,J156,J157,J158,J1581,J1588,J159,J16,J160,J168,J17,J170,J171,J172,J173,J178,J18,J180,J181,J182,J188,J189,J20,J200,J201,J202,J203,J204,J205,J206,J207,J208,J209,J21,J210,J211,J218,J219, |
| 44 | Chronic obstructive pulmonary disease | J41,J410,J411,J418,J42,J43,J430,J431,J432,J438,J439,J44,J440,J441,J442,J448,J449, |
| 45 | Arrhythmia | I440,I441,I442,I443,I444,I445,I446,I447,I45,I450,I451,I452,I453,I454,I455,I456,I458,I459,I46,I460,I461,I469,I47,I470,I471,I472,I479,I48,I49,I490,I4900,I4901,I4908,I4909,I491,I492,I493,I494,I495,I498,I499, |
| 46 | Metabolic disorder | E870,E871,E872,E873,E874,E875,E876,E878,E88, |
| 47 | Gastrointestinal bleeding | K920,K921,K922, |
| 48 | Valvular heart disease and cardiomyopathy | I34,I340,I341,I342,I348,I349,I35,I350,I351,I352,I358,I359,I36,I360,I361,I362,I368,I369,I37,I370,I371,I372,I378,I379,I38,I39,I390,I391,I392,I393,I394,I398,I40,I400,I401,I408,I409,I41,I410,I411,I412,I418,I42,I420,I421,I422,I423,I424,I425,I426,I427,I428,I429,I43,I430,I431,I432,I438,I514,I511,I05,I050,I051,I052,I058,I059,I06,I060,I061,I062,I068,I069,I07,I070,I071,I072,I078,I079,I08,I080,I081,I082,I083,I088,I089,I09,I090,I091,I092,I098,I099, |
| 49 | Cerebrovascular disease | I63,I630,I631,I632,I633,I634,I635,I636,I638,I639,I64,I693,G45,G450,G451,G452,G453,G454,G458,G459,G46,G460,G461,G462,G463,G464,G465,G466,G467,G468,Z867,I60,I600,I601,I602,I603,I604,I605,I606,I607,I608,I609,I61,I610,I611,I612,I613,I614,I615,I616,I618,I619,I62,I620,I621,I629,I670,I671,I672,I673,I674,I675,I676,I677,I678,I679,I68,I680,I681,I682,I688,I69,I690,I691,I692,I694,I698, |
| 50 | Gastrointestinal tract infection | A000,A009,A010,A011,A020,A021,A022,A029,A031,A039,A042,A044,A047,A048,A049,A050,A058,A059,A060,A069,A071,A078,A079,A082,A083,A084,A085,A09,A090,A099, |
| 51 | Other infectious diseases | A150,A151,A152,A153,A154,A156,A157,A159,A160,A161,A162,A164,A165,A167,A169,A170,A178,A179,A180,A181,A182,A183,A184,A188,A190,A191,A192,A198,A199,A227,A241,A242,A243,A244,A270,A278,A279,A308,A319,A35,A360,A390,A398,A400,A401,A402,A403,A408,A409,A410,A411,A412,A413,A414,A415,A418,A419,A430,A441,A449,A46,A480,A490,A493,A498,A499,A510,A521,A523,A528,A529,A540,A542,A590,A600,A630,A690,A719,A752,A753,A759,A799,A86,A881,A90,A91,A910,A911,A919,A920,B001,B002,B003,B004,B005,B008,B009,B018,B019,B020,B022,B023,B027,B028,B029,B052,B059,B09,B169,B171,B178,B179,B181,B1819,B182,B189,B199,B200,B201,B202,B203,B204,B205,B206,B207,B208,B209,B212,B218,B220,B221,B222,B227,B230,B231,B232,B238,B24,B269,B279,B309,B332,B338,B348,B349,B354,B370,B371,B376,B377,B379,B382,B440,B449,B450,B451,B459,B460,B461,B479,B484,B500,B508,B509,B519,B54,B571,B59,B650,B660,B669,B690,B699,B769,B770,B779,B780,B789,B829,B832,B89,B908,B909,B99,L00,L010,L020,L021,L022,L023,L024,L028,L029,L030,L031,L032,L033,L038,L039,L040,L041,L042,L043,L048,L049,L080,L088,L089,N390, |
| 52 | Other cardiovascular diseases | I00,I011,I012,I019,I020,I10,I119,I120,I129,I131,I139,I150,I151,I152,I158,I159,I260,I269,I270,I271,I272,I278,I279,I280,I288,I300,I301,I309,I311,I312,I313,I318,I319,I330,I339,I510,I513,I515,I516,I517,I518,I519,I651,I652,I658,I659,I660,I661,I662,I663,I664,I668,I669,I700,I701,I702,I7021,I709,I710,I711,I712,I713,I714,I716,I719,I720,I722,I723,I724,I728,I729,I730,I731,I738,I739,I740,I741,I742,I743,I744,I745,I748,I749,I770,I771,I772,I775,I776,I778,I779,I781,I800,I801,I802,I803,I808,I809,I81,I820,I822,I823,I828,I829,I830,I839,I840,I841,I842,I843,I844,I845,I847,I848,I849,I850,I859,I864,I870,I871,I872,I878,I879,I881,I889,I890,I891,I950,I951,I952,I958,I959,I970,I971,I978,I99, |
| 53 | Diabetes mellitus and its complication | E100,E101,E102,E104,E105,E106,E107,E108,E109,E110,E111,E112,E113,E114,E115,E116,E117,E118,E119,E130,E139,E140,E141,E142,E143,E145,E147,E149,E15,E160,E161,E162,E169, |

# **Table S2**. Parameters associated with in-hospital mortality for all heart failure hospitalizations (N=434,933) in Thailand between 2008-2013

| **Parameter** | **n of in-hospital deaths / N of all hospitalizations in group**  **(%)** | **Crude odds ratio**  **(95% CI)** | ***P*-value** |
| --- | --- | --- | --- |
| **Overall** | 17,922 / 434,933  (4.1) |  | - |
| **Age group, y** |  |  |  |
| **18-39** | 1,317 / 25,079  (5.3) | Ref. |  |
| **40-59** | 4,376 / 117,873  (3.7) | 0.70 (0.65-0.74) | < 0.001 |
| **60-79** | 8,750 / 224,566  (3.9) | 0.73 (0.69-0.78) | < 0.001 |
| **≥ 80** | 3,479 / 67,415 (5.2) | 0.98 (0.92-1.05) | 0.580 |
| **Female** | 10,223 / 252,648  (4.0) | Ref. |  |
| **Male** | 7,699 / 182,285  (4.2) | 1.05 (1.02-1.08) | 0.004 |
| **Comorbidities** |  |  |  |
| **Prior HF** | 8,326 / 206,220  (4.0) | 0.96 (0.93-0.99) | 0.009 |
| **Prior MI** | 1,954 / 46,722  (4.2) | 1.02 (0.97-1.07) | 0.479 |
| **CAD** | 7,005 / 164,797  (4.3) | 1.05 (1.02-1.09) | 0.001 |
| **Non-rheumatic VHD** | 3,116 / 69,679  (4.5) | 1.11(1.07-1.53) | < 0.001 |
| **Rheumatic VHD** | 2,133 / 44,619  (4.8) | 1.19 (1.14-1.25) | < 0.001 |
| **Cardiomyopathy and myocarditis** | 1,652 / 35,581  (4.6) | 1.15 (1.09-1.21) | < 0.001 |
| **Congenital heart disease** | 279 / 4,943  (5.6) | 1.40 (1.24-1.58) | < 0.001 |
| **AF/AFL** | 4,635 / 103,700  (4.5) | 1.12 (1.08-1.16) | < 0.001 |
| **Conduction abnormality** | 814 / 18,166  (4.5) | 1.10 (1.02-1.18) | 0.013 |
| **Ischemic stroke** | 1,151 / 18,229  (6.3) | 1.61 (1.51-1.71) | < 0.001 |
| **Other CVD** | 1,003 / 16,787  (6.0) | 1.51 (1.41-1.61) | < 0.001 |
| **PAD and disease of aorta** | 142 / 2,672  (5.3) | 1.31 (1.10-1.55) | 0.002 |
| **Hypertension** | 9,475 / 262,379  (3.6) | 0.73 (0.71-0.75) | < 0.001 |
| **Diabetes mellitus** | 5,859 / 153,793  (3.8) | 0.88 (0.86-0.91) | < 0.001 |
| **Dyslipidemia** | 4,307 / 134,581  (3.2) | 0.70 (0.67-0.72) | < 0.001 |
| **Renal failure** | 9,465 / 156,324  (6.1) | 2.06 (2.0-2.12) | < 0.001 |
| **COPD** | 2,317 / 53,120  (4.4) | 1.07 (1.02-1.12) | 0.003 |
| **Liver disease** | 2,007 / 23,343  (8.6) | 2.34 (2.23-2.46) | < 0.001 |
| **Depression** | 153 / 3,693  (4.1) | 1.01 (0.86-1.18) | 0.945 |
| **Dementia** | 96 / 1,231  (7.8) | 1.97 (1.60-2.43) | < 0.001 |
| **HIV** | 251 / 2,855  (8.8) | 2.26 (1.98-2.58) | < 0.001 |
| **Thalassemia** | 368 / 7,491  (4.9) | 1.21 (1.09-1.34) | 0.001 |
| **Complications** |  |  |  |
| **VT/VF** | 601 / 1,645  (36.5) | 13.8 (12.5-15.3) | < 0.001 |
| **Pneumonia** | 3,513 / 25,194  (13.9) | 4.45 (4.27-4.62) | < 0.001 |
| **Acute kidney injury** | 4,273 / 25,068  (17.0) | 5.97 (5.75-6.19) | < 0.001 |
| **Hyponatremia** | 2,681 / 29,378  (9.1) | 2.57 (2.46-2.68) | < 0.001 |
| **Anemia** | 4,020 / 77,318  (5.2) | 1.36 (1.31-1.41) | < 0.001 |
| **Procedure** |  |  |  |
| **Ventilator** | 10,519 / 34,776  (30.2) | 23.0 (22.3-23.9) | < 0.001 |
| **Echocardiography** | 1,272 / 32,269  (3.9) | 0.95 (0.90-1.01) | 0.093 |
| **Cardiac catheterization** | 52 / 2,714  (1.9) | 0.45 (0.34-0.60) | < 0.001 |
| **Renal replacement therapy** | 593 / 3,235  (18.3) | 5.37 (4.90-5.88) | < 0.001 |
| **Cardioversion/defibrillation** | 737 / 1161  (63.5) | 42.1 (37.5-47.5) | < 0.001 |

Abbreviations: AF/AFL, atrial fibrillation or atrial flutter; CAD, coronary artery disease; COPD, chronic obstructive pulmonary disease; CSMBS, Civil Servant Medical Benefit scheme; HIV, human immunodeficiency virus infection; HF, heart failure; CVD, cerebrovascular disease; MI, myocardial infarction; PAD, peripheral arterial disease; Ref., reference category; SSS, Social Security scheme; UCS, Universal Health Coverage scheme; VHD, valvular heart disease; VT/VF, ventricular tachycardia or ventricular fibrillation

Notes:- Unconditional binary logistic regression analysis was used for univariable analysis.

# **Table S3**. Parameters associated with 30-day heart failure rehospitalization (N = 378,031) in Thailand between 2008 and 2013^a^

| **Parameter** | **n of 30-d HF rehospitalization / N of all patients discharged alive, HF rehospitalization rate**  **(%)** | **Crude odds ratio**  **(95% CI)** | ***P*-value** |
| --- | --- | --- | --- |
| **Overall** | 62,056 / 378,031  (16.4) |  |  |
| **Age group, y** |  |  |  |
| **18-39** | 4,357 / 21,042  (20.7) | Ref. |  |
| **40-59** | 18,468 / 102,706  (18.0) | 0.84 (0.81-0.87) | < 0.001 |
| **60-79** | 31,853/ 195,955  (16.3) | 0.74 (0.72-0.77) | < 0.001 |
| **≥ 80** | 7,378/ 58,328  (12.6) | 0.56 (0.53-0.58) | < 0.001 |
| **Female** | 34,302 / 220,514  (15.6) | Ref. |  |
| **Male** | 27,754 / 157,517  (17.6) | 1.16 (1.14-1.18) | < 0.001 |
| **Comorbidities** |  |  |  |
| **Prior HF** | 44,522 / 180,706  (24.6) | 3.35 (3.29-3.42) | < 0.001 |
| **Prior MI** | 10,495 / 40,818  (25.7) | 1.92 (1.87-1.96) | < 0.001 |
| **CAD** | 30,902 / 144,088  (21.4) | 1.78 (1.75-1.81) | < 0.001 |
| **Non-rheumatic VHD** | 14,530 / 59,771  (24.3) | 1.83 (1.79-1.87) | < 0.001 |
| **Rheumatic VHD** | 9,358 / 38,318  (24.4) | 1.76 (1.72-1.81) | < 0.001 |
| **Cardiomyopathy and myocarditis** | 8,448 / 31,025  (27.2) | 2.05 (1.99-2.10) | < 0.001 |
| **Congenital heart disease** | 986 / 4,140  (23.8) | 1.60 (1.49-1.72) | < 0.001 |
| **AF/AFL** | 17,423 / 90,025  (19.4) | 1.31 (1.28-1.33) | < 0.001 |
| **Conduction abnormality** | 3,166 / 15,531  (20.4) | 1.32 (1.27-1.37) | < 0.001 |
| **Ischemic stroke** | 2,978 / 15,302  (19.5) | 1.24 (1.19-1.29) | < 0.001 |
| **Other CVD** | 2,773 / 14,278  (19.4) | 1.24 (1.19-1.29) | < 0.001 |
| **PAD and disease of aorta** | 538 / 2,315  (23.2) | 1.55 (1.40-1.70) | < 0.001 |
| **Hypertension** | 40,231 / 230,628  (17.4) | 1.22 (1.19-1.24) | < 0.001 |
| **Diabetes mellitus** | 24,531 / 134,491  (18.2) | 1.23 (1.20-1.25) | < 0.001 |
| **Dyslipidemia** | 23,300 / 119,713  (19.5) | 1.37 (1.35-1.39) | < 0.001 |
| **Renal failure** | 25,750 / 130,289  (19.8) | 1.43(1.41-1.46) | < 0.001 |
| **COPD** | 9,289 / 45,925  (20.2) | 1.34 (1.31-1.38) | < 0.001 |
| **Liver disease** | 4,255 / 18,736  (22.7) | 1.53 (1.48-1.59) | < 0.001 |
| **Depression** | 835 / 3,217  (26.0) | 1.80 (1.66-1.94) | < 0.001 |
| **Dementia** | 162 / 1,048  (15.5) | 0.93 (0.79-1.10) | 0.402 |
| **HIV** | 567 / 2,352  (24.1) | 1.62 (1.48-1.79) | < 0.001 |
| **Thalassemia** | 1,272 / 6,378  (19.9) | 1.27 (1.20-1.36) | < 0.001 |
| **Complications** |  |  |  |
| **VT/VF** | 119 / 836  (14.2) | 0.85 (0.70-1.03) | 0.089 |
| **Pneumonia** | 1,957 / 17,690  (11.1) | 0.62(0.59-0.65) | < 0.001 |
| **Acute kidney injury** | 2,566 / 17,173  (14.9) | 0.89 (0.85-0.93) | < 0.001 |
| **Hyponatremia** | 3,953 / 23,084  (17.1)) | 1.06 (1.02-1.09) | 0.003 |
| **Anemia** | 9,674 / 65,993  (14.7) | 0.85 (0.83-0.87) | < 0.001 |
| **Procedure** |  |  |  |
| **Ventilator** | 2,343 / 21,600  (10.8) | 0.61 (0.58-0.63) | < 0.001 |
| **Echocardiography** | 3,368 / 28,886  (11.7) | 0.65 (0.63-0.68) | < 0.001 |
| **Cardiac catheterization** | 238/ 2,502  (9.5) | 0.53 (0.47-0.61) | < 0.001 |
| **Renal replacement therapy** | 138 / 2,496  (5.5) | 0.30(0.25-0.35) | < 0.001 |
| **Cardioversion / defibrillation** | 27 / 335  (8.1) | 0.45(0.30-0.66) | < 0.001 |

Abbreviations: AF/AFL, atrial fibrillation or atrial flutter; CAD, coronary artery disease; COPD, chronic obstructive pulmonary disease; CSMBS, Civil Servant Medical Benefit scheme; HIV, human immunodeficiency virus infection; HF, heart failure; CVD, cerebrovascular disease; MI, myocardial infarction; PAD, peripheral arterial disease; Ref., reference category; SSS, Social Security scheme; UCS, Universal Health Coverage scheme; VHD, valvular heart disease; VT/VF, ventricular tachycardia or ventricular fibrillation

^a^ Excluding patients discharged in December 2013

Notes:- Unconditional binary logistic regression analysis was used for univariable analysis.

#

# **Table S4.** Parameters associated with one-year mortality for all hospitalization (N=246,958) between 2008 and 2012 in Thailand

| **Prognostic factor** | **n of deaths / N of total patients in group**  **(one-year mortality rate, %)** | **Crude odds ratio**  **(95% CI)** | ***P*-value** |
| --- | --- | --- | --- |
| **Overall** | 74,266 / 246,958  (30.1) |  |  |
| **Age group, y** |  |  |  |
| **18-39** | 3,499 / 14,027  (24.9) | Ref. |  |
| **40-59** | 16,305 / 65,968  (24.7) | 0.99 (0.95-1.03) | 0.570 |
| **60-79** | 38,883 / 127,067  (30.6) | 1.33 (1.28-1.38) | < 0.001 |
| **≥ 80** | 15,579 / 39,900  (39.1) | 1.93 (1.85-2.01) | < 0.001 |
| **Female** | 42,011 / 144,844  (29.0) | Ref. | - |
| **Male** | 32,255 / 102,118  (31.6) | 1.13 (1.11-1.15) | < 0.001 |
| **Comorbidities** |  |  |  |
| **Prior HF** | 29,061 / 81,267  (35.8) | 1.48 (1.46-1.51) | < 0.001 |
| **Prior MI** | 7,712 / 20,481  (37.7) | 1.45 (1.41-1.50) | < 0.001 |
| **CAD** | 25,896 / 81,097  (31.9) | 1.14 (1.12-1.16) | < 0.001 |
| **Non-rheumatic VHD** | 10,157 / 31,316  (32.4) | 1.14 (1.11-1.16) | < 0.001 |
| **Rheumatic VHD** | 6,417 / 20,361  (31.5) | 1.08 (1.04-1.11) | < 0.001 |
| **Cardiomyopathy and myocarditis** | 4,734 / 14,287  (33.1) | 1.16 (1.12-1.21) | < 0.001 |
| **Congenital heart disease** | 744 / 2,287  (32.5) | 1.12 (1.03-1.23) | 0.01 |
| **AF/AFL** | 15,586 / 51,949  (30.0) | 1.00 (0.98-1.02) | 0.698 |
| **Conduction abnormality** | 2,753 / 8,871  (31.0) | 1.05 (1.01-1.10) | 0.044 |
| **Ischemic stroke** | 3,663 / 9,480  (38.6) | 1.49 (1.43-1.55) | < 0.001 |
| **Other CVD** | 3,374 / 8,628  (39.1) | 1.52 (1.45-1.59) | < 0.001 |
| **PAD and disease of aorta** | 551 / 1,302  (42.3) | 1.71 (1.53-1.91) | < 0.001 |
| **Hypertension** | 41,295 / 139,429  (29.6) | 0.95 (0.94-0.97) | < 0.001 |
| **Diabetes mellitus** | 25,191 / 81,810  (30.8) | 1.05 (1.03-1.07) | < 0.001 |
| **Dyslipidemia** | 17,595 / 65,772  (26.8) | 0.80 (0.79-0.82) | < 0.001 |
| **Renal failure** | 33,430 / 78,886  (42.4) | 2.29 (2.25-2.33) | < 0.001 |
| **COPD** | 10,935 / 26,982  (40.5) | 1.69 (1.64-1.73) | < 0.001 |
| **Liver disease** | 5,571 / 11,582  (48.1) | 2.25 (2.17-2.34) | < 0.001 |
| **Depression** | 512 / 1,523  (33.6) | 1.18 (1.06-1.31) | 0.002 |
| **Dementia** | 278 / 652  (42.6) | 1.73 (1.48-2.02) | < 0.001 |
| **HIV** | 690 / 1,481  (46.6) | 2.04 (1.84-2.56) | < 0.001 |
| **Thalassemia** | 1,359 / 3,776  (36.0) | 1.31 (1.23-1.40) | < 0.001 |
| **Complications** |  |  |  |
| **VT/VF** | 615 / 987  (62.3) | 3.87 (3.40-4.40) | < 0.001 |
| **Pneumonia** | 7,030 / 15,360  (45.8) | 2.06 (2.00-2.13) | < 0.001 |
| **Acute kidney injury** | 6,822 / 13,794  (49.5) | 2.40 (2.32-2.49) | < 0.001 |
| **Hyponatremia** | 7,323 / 16,532  (44.3) | 1.94 (1.88-2.01) | < 0.001 |
| **Anemia** | 17,926 / 45,977  (39.0) | 1.64 (1.61-1.68) | < 0.001 |
| **Procedure** |  |  |  |
| **Ventilator** | 12,685 / 21,561  (58.8) | 3.80 (3.69-3.91) | < 0.001 |
| **Echocardiography** | 4,755 / 19,140  (24.8) | 0.75 (0.73-0.78) | < 0.001 |
| **Cardiac catheterization** | 237 / 1,487  (15.9) | 0.44 (0.38-0.51) | < 0.001 |
| **Renal replacement therapy** | 932 / 2,120  (44.0) | 1.84 (1.68-2.00) | < 0.001 |
| **Cardioversion/defibrillation** | 147 / 192  (82.1) | 10.8 (8.92-13.0) | < 0.001 |

Abbreviations: AF/AFL, atrial fibrillation or atrial flutter; CAD, coronary artery disease; COPD, chronic obstructive pulmonary disease; CSMBS, Civil Servant Medical Benefit scheme; HIV, human immunodeficiency virus infection; HF, heart failure; CVD, cerebrovascular disease; MI, myocardial infarction; PAD, peripheral arterial disease; Ref., reference category; SSS, Social Security scheme; UCS, Universal Health Coverage scheme; VHD, valvular heart disease; VT/VF, ventricular tachycardia or ventricular fibrillation

Notes:- Unconditional binary logistic regression analysis was used for univariable analysis.
